# Synthesis of some new pyrazolo[1,5-*a*]pyrimidine, pyrazolo[5,1-*c*]triazine, 1,3,4-thiadiazole and pyridine derivatives containing 1,2,3-triazole moiety

**DOI:** 10.1186/s13065-017-0282-4

**Published:** 2017-06-12

**Authors:** Nadia A. Abdelriheem, Yasser H. Zaki, Abdou O. Abdelhamid

**Affiliations:** 1 Department of Chemistry, Faculty of Science, Cairo University, Giza, 12613 Egypt; 2 Department of Chemistry, Faculty of Science, Beni-Suef University, Beni-Suef, 62514 Egypt; 3 Department of Chemistry, Faculty of Science and Humanity Studies at Al-Quwayiyah, Shaqra University, Al-Quwayiyah, 11971 Saudi Arabia

**Keywords:** 1,2,3-Triazole, Pyrazolo[1,5-*a*]pyrimidines, Pyrazolo[5,1-*c*]triazines, Thieno[2,3-*b*]pyridines, 1,3,4-Thiadiazoles, Hyrazonoyl chlorides, Thiazoles, Pyridines

## Abstract

**Background:**

Pyrazolo[1,5-*a*]pyrimidines are purine analogues. They have beneficial properties as antimetabolites in purine biochemical reactions. This division compounds have attracted wide pharmaceutical interest because of their antitrypanosomal activity.

**Results:**

The present work depicts an effective synthesis convention of pyrazolo[1,5-*a*]pyrimidines, pyrazolo[5,1-*c*]triazines, thieno[2,3-*b*]pyridines and polysubstituted pyridines containing 1,2,3,-triazole moiety from the reaction of sodium 3-(5-methyl-1-(*p*-toly)-1*H*-1,2,3-triazol-4-yl)-3-oxoprop-1-en-1-olate with the fitting heterocyclic amines and its diazonium salt, and active methylene compounds, individually. Likewise, thiazoles and, 1,3,4-thiadiazoles were obtained from 2-bromo-1-(5-methyl-1-(*p*-tolyl)-1*H*-1,2,3-triazol-4-yl)ethanone and some reagent such as hydrazonoyl chlorides and halo ketones. The newly synthesized compounds were established by elemental analysis, spectral data, and alternative synthetic route whenever possible.

**Conclusions:**

New series of pyrazolo[1,5-*a*]pyrimidines, pyrazolo[5,1-*c*]triazines, thieno[2,3-*b*]pyridines and polysubstituted pyridines containing the 1,2,3,-triazole moiety were synthesized via reactions of sodium 3-(5-methyl-1-(*p*-toly)-1*H*-1,2,3-triazol-4-yl)-3-oxoprop-1-en-1-olate with the appropriate heterocyclic amines and its diazonium salt. In addition, 1,3,4-thiadiazoles and, 1,3-thiazoles were acquired in a decent yield via the reaction of substituted thiourea with the appropriate hydrazonoyl chlorides and halogenated ketenes.

**Graphical abstract:**
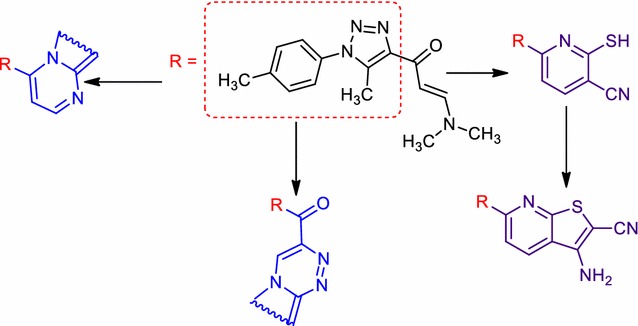
Synthesis of some new pyrazolo[1,5-*a*]pyrimidines, pyrazolo[5,1-*c*]triazines and thieno[2,3-*b*]pyridines.

## Background

Pyrazolo[1,5-*a*]pyrimidines are purine analogs and therefore have valuable properties as antimetabolites in purine biochemical activity. This class of compounds has attracted wide pharmaceutical interest because of their antitrypanosomal activity [1], antischistosomal activity [2], and other activities such as HMG-CoA reductase inhibitors [3], COX-2 selective inhibitors [4], AMP phosphodiesterase inhibitors [5], KDR kinase inhibitors [6], selective peripheral benzodiazepine receptor ligaments [7], antimicrobial agents [8], and as antianxiety agents [9]. Recently other pharmaceutical activities have been reported, for example, as an agent for the treatment of sleep disorders [10] and as an oncological agent [6]. Also, pyrazolo[5,1-*c*][1,2,4]triazines are known to exhibit a broad range of biological activities [11–15]. Due to their structural similarities to nucleic bases, pyrazolo[5,1-*c*][1,2,4]triazines may act as metabolites and therefore they can be useful as antiviral and antitumor agents [11]. Pyrazolotriazines have indicated a remarkable cytotoxic activity against colon, breast, and lung carcinoma cells [16]. Some derivatives showed selective cytotoxicity in hypoxic and normoxic conditions [17]. The 1,3,4-thiadiazole derivatives have attracted considerable interest due to their wide spectra of biological activities such as antibacterial, antifungal, antituberculosis, anti-hepatitis B viral, antileishmanial, anti-inflammatory, analgesic, CNS depressant, anticancer, antioxidant, antidiabetic, molluscicidal, antihypertensive, diuretic, analgesic, antimicrobial, antitubercular, and anticonvulsant activities [18–27].

## Results and discussion

### Chemistry

**Scheme 1:**
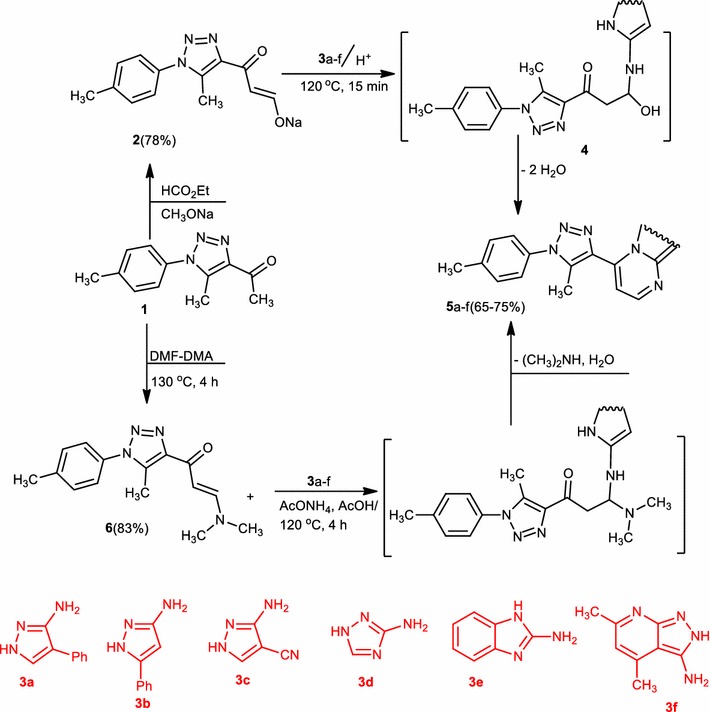
Synthesis of pyrazolo[1,5-*a*]pyrimidines (**5a**–**c**), 1,2,4-triazolo[1,5-*a*]pyrimidine (**5d**), benzo [4,5]imidazo[1,2-*a*]pyrimidine (**5e**), and pyrido[2′,3′:3,4]pyrazolo[1,5-*a*]pyrimidine (**5f**)

The reaction of 1-(5-methyl-1-(*p*-tolyl)-1*H*-1,2,3-triazol-4-yl)ethan-1-one (**1**) with ethyl formate in diethyl ether in the presence of sodium methoxide has afforded sodium 3-(5-methyl-1-(*p*-tolyl)-1*H*-1,2,3-triazol-4-yl)-3-oxoprop-1-en-1-olate (**2**). Likewise, compound (**1**) reacted with *N,N*-dimethylformamide-dimethylacetal in boiling xylene to afford 3-(dimethylamino)-1-(5-methyl-1-(*p*-tolyl)-1*H*-1,2,3-triazol-4-yl)prop-2-en-1-one (**6**). The reactivity of compound (**2**) and compound (**6**) towards heterocyclic amines was inspected. In this manner, reaction of compound (**2**) or compound (**6**) with each of 3-amino-5-phenylpyrazole (**3a**), 3-amino-4-phenylpyrazole (**3b**), 3-amino-4-cyanopyrazole (**3c**), 3-amino-1,2,4-triazole (**3d**), 2-aminobenzimidazole (**3e**) and 4,6-dimethyl-2*H*-pyrazolo[3,4-*b*]pyridin-3-amine (**3f**) in refluxing piperidinium acetate, in each case, only one isolable product as evidenced by *TLC.* The isolated products (**5a**–**f**) (Scheme 1) were identified, on the base of their elemental analysis, spectral data and according to similar data obtained before [28–30].

**Scheme 2:**
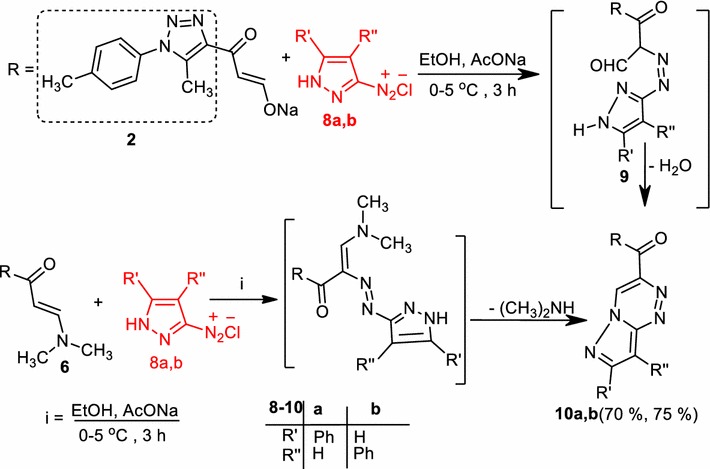
Synthesis of pyrazolo[5,1-*c*]triazines (**10**)

The reaction of compound (**2**) or compound (**6**) with each of diazotized 3-amino-5-phenylpyrazole (**8a**) and diazotized 3-amino-4-phenylpyrazole (**8b**) in ethanol containing sodium acetate at 0–5 °C yielded products that were distinguished as (5-methyl-1-(*p*-tolyl)-1*H*-1,2,3-triazol-4-yl)(7-phenylpyrazolo[5,1-*c*][1,2,4]triazin-3-yl)-methanone (**10a**) and (5-methy-1-(*p*-tolyl)-1*H*-1,2,3-triazol-4-yl)(8-phenylpyrazolo[5,1-*c*][1,2,4]triazin-3-yl)-methanone (**10b**), respectively (Scheme 2). The structures of the products (**10a**) and (**10b**) were consistent with their elemental and spectral (Ms, IR, ^1^H NMR, and the ^13^C NMR) analysis (see “Experimental section”). To account for the formation of the products **10a** and **10b**, it is suggested as depicted in (Scheme 2) that the reaction start with electrophilic substitution to yield the corresponding azo derivative, which undergoes in situ dehydrative cyclization, gave the corresponding **10** as a final product.

**Scheme 3:**
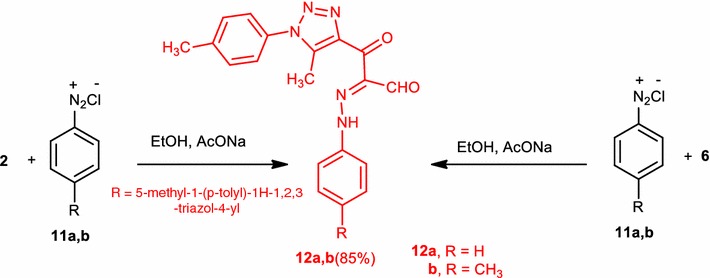
Synthesis of 3-(5-methyl-1-(*p*-tolyl)-1*H*-1,2,3-triazol-4-yl)-3-oxo-2-(2-(aryl)hydrazono)propanal (**12a**) and (**12b**)

Treatment of compound (**2**) with each of benzenediazonium chloride (**11a**) or *p*-toluidine diazonium chloride (**11b**) in ethanol containing sodium acetate as a buffer solution yielded 3-(5-methyl-1-(*p*-tolyl)-1*H*-1,2,3-triazol-4-yl)-3-oxo-2-(2-phenylhydrazono)propanal (**12a**), 3-(5-methyl-1-(*p*-tolyl)-1*H*-1,2,3-triazol-4-yl)-3-oxo-2-(2-(*p*-tolyl)hydrazono)propanal (**12b**), respectively (Scheme 3). The structures of compound (**12a**) and compound (**12b**) were affirmed by elemental analysis, spectral data, and alternative synthetic route. In this way, 3-(dimethylamino)-1-(5-methyl-1-(*p*-tolyl)-1*H*-1,2,3-triazol-4-yl)prop-2-en-1-one (**6**) was coupled with benzenediazonium chloride or *p*-toluidinediazonium chloride to give a product indistinguishable in all aspects (m.p., mixed m.p. and spectra) with compound (**12a**) and compound (**12b**), respectively. The ^1^H NMR spectrum of compound (**12a**) showed signals at δ = 2.06 (s, 3H, CH_3_), 2.34 (s, 3H, 4-CH_3_C_6_H_4_), 7.26–8.20 (m, 9H, ArH’s), 9.75 (s, 1H, CHO) and 14.39 (s, br., NH).

**Scheme 4:**
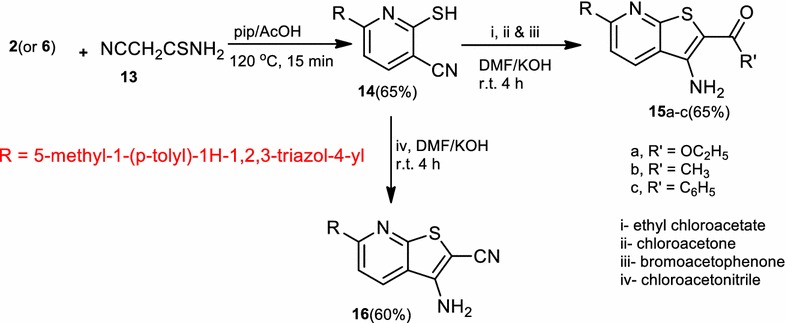
Synthesis of thieno[2,3-*b*]pyridines (**15a**–**c**) and (**16**)

Reaction of compound (**2**) with cyanothioacetamide (**13**) in piperdinium acetate gave 2-mercapto-6-(5-methyl-1-(*p*-tolyl)-1*H*-1,2,3-triazol-4-yl)nicotinonitrile (**14**). The Structure of compound (**14**) was elucidated by elemental analysis, spectral data, and alternative synthetic route or chemical transformation. Thus, treatment of compound (**6**) with cyanothioacetamide in ethanol containing a catalytic amount of piperidine under reflux gave a product identical in all aspects (m.p., mixed m.p. and spectra) with compound (**14**). The product formulated from treatment of compound (**14**) with ethyl chloroacetate, in *N,N*-dimethylformamide containing potassium hydroxide was ethyl 3-amino-6-(5-methyl-1-(*p*-tolyl)-1*H*-1,2,3-triazol-4-yl)thieno[2,3-*b*]pyridine-2-carboxylate (**15a**) corresponding to the addition, dehydrochlorination, and cyclization reactions (Scheme 4). IR spectrum of compound (**15a**) showed a band at 3460, 3355 (NH_2_ group) and no band of the CN function between 2100 and 2300 cm^−1^. The ^1^H NMR spectrum of compound (**15a**) revealed signals at 1.26 (t, 3H, *J* = 7 Hz, CH_2_CH_3_), 2.34 (s, 3H, 4-CH_3_C_6_H_4_), 2.64 (s, 3H, CH_3_), 4.23 (q, 2H, *J* = 7 Hz, CH_2_CH_3_), 6.8 (s, br., 2H, NH_2_), 7.32–7.63 (m, 5H, ArH’s) and 8.81–8.83 (d, 1H, ArH) and absence of signals of the –SCH_2_– group. These results proved that the CN and the –SCH_2_– groups were both involved in the cyclization step leading to compound (**15a**).

**Scheme 5:**
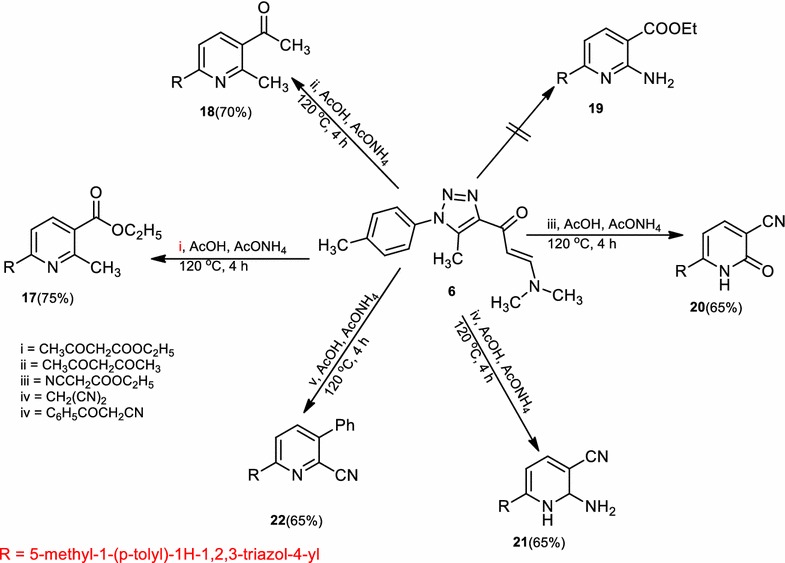
Synthesis of pyridine derivatives (**17**), (**18**), and (**20**–**22**)

Also, compound (**14**) was reacted with each of chloroacetone and ω-bromoacetophenone in *N,N*-dimethylformamide containing potassium hydroxide to afford 1-(3-amino-6-(5-methyl-1-(*p*-tolyl)-1*H*-1,2,3-triazol-4-yl)thieno[2,3-*b*]pyridin-2-yl)ethan-1-one (**15b**) and 6-(3-amino-6-(5-methyl-1-(*p*-tolyl)-1*H*-1,2,3-triazol-4-yl)thieno[2,3-*b*]pyridin-2-yl)(phenyl)methanone (**15c**) respectively. Similarly, compound (**14**) was reacted with chloroacetonitrile afforded 3-amino-6-(5-methyl-1-(*p*-tolyl)-1*H*-1,2,3-triazol-4-yl)thieno[2,3-*b*]pyridine-2-carbonitrile (**16**), in a good yield (Scheme 4). The structures of compounds (**15a**–**c**) and (**16**) were confirmed by elemental analysis and spectral data. Treatment of compound (**6**) with each of ethyl acetoacetate, acetylacetone, ethyl cyanoacetate, malononitrile or benzoylacetonitrile in boiling acetic acid containing ammonium acetate under reflux gave ethyl 2-methyl-6-(5-methyl-1-*p*-tolyl-1*H*-1,2,3-triazol-4-yl)pyridine-3-carboxylate (**17**), 1-(2-methyl-6-(5-methyl-1-*p*-tolyl-1*H*-1,2,3-triazol-4-yl)pyridin-3-yl)ethanone (**18**), 1,2-dihydro-6-(5-methyl-1-*p*-tolyl-1*H*-1,2,3-triazol-4-yl)-2-oxopyridine-3-carbonitrile (**20**), 2-amino-6-(5-methyl-1-*p*-tolyl-1*H*-1,2,3-triazol-4-yl)pyridine-3-carbonitrile (**21**), 6-(5-methyl-1-(*p*-tolyl)-1*H*-1,2,3-triazol-4-yl)pyridin-3-phenyl-2-carbonitrile (**22**), respectively (Scheme 5). Structures (**17**), (**18**), and (**20**–**22**) were confirmed based on elemental analysis and spectral data (cf. “Experimental section”).

**Scheme 6:**
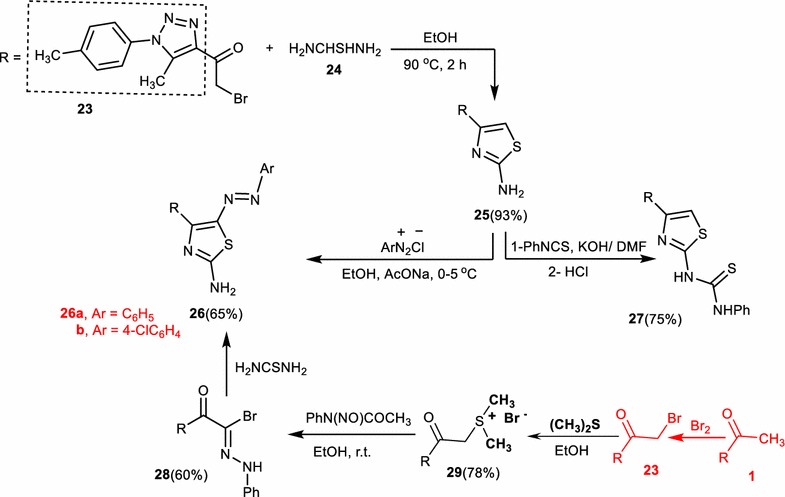
Synthesis of thiazoles (**25**), (**26**), and (**27**)

Next, 4-(5-methyl-1-(*p*-tolyl)-1*H*-1,2,3-triazol-4-yl)thiazol-2-amine (**25**) was prepared from the reaction of 2-bromo-1-(5-methyl-1-(*p*-tolyl)-1*H*-1,2,3-triazol-4-yl)ethanone (**23**) [31] with thiourea. The structure of compound (**25**) was established based on elemental analysis, spectral data, and chemical transformation. Thus, compound (**25**) was coupled with arenediazonium chlorides in ethanol contained sodium acetate to afford 4-(5-methyl-1-(*p*-tolyl)-1*H*-1,2,3-triazol-4-yl)-5-(phenyldiazenyl)thiazol-2-amine (**26a**) and 5-((4-chlorophenyl)diazenyl)-4-(5-methyl-1-(*p*-tolyl)-1*H*-1,2,3-triazol-4-yl)thiazol-2-amine (**26b**), respectively (Scheme 6). More evidence on the correct structure of compound (**26a**) was obtained via reaction of thiourea with 2-(5-methyl-1-(*p*-tolyl)-1*H*-1,2,3-triazol-4-yl)-2-oxo-*N*-phenylacetohydrazonoyl bromide (**28**) in boiling ethanol (cf. “Experimental section”).

**Scheme 7:**
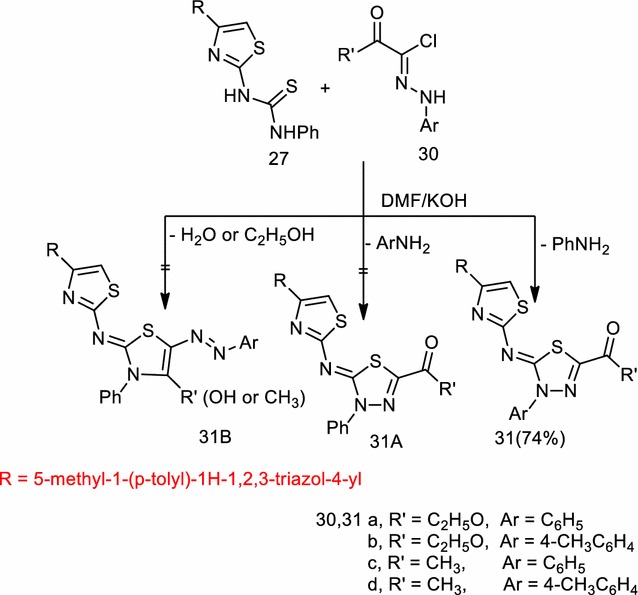
Synthesis of 1,3,4-thiadiazoles (**31a**–**d**)

1-(4-(5-Methyl-1-(*p*-tolyl)-1*H*-1,2,3-triazol-4-yl)thiazol-2-yl)-3-phenylthiourea (**27**) was prepared via reaction of compound (**25**) with phenyl isothiocyanate in *N,N*-dimethylformamide containing potassium hydroxide, followed by acidification with hydrochloric acid. The structure of compound (**27**) was confirmed by elemental analysis, spectral data, and chemical transformation. Thus, the appropriate hydrazonoyl chloride (**30a**–**d**) were reacted with thioanilide (**27**) in *N,N*-dimethylformamide in presence of triethylamine or potassium hydroxide to give one isolable product according to TLC. The structure of the product may be one from the structure of compound (**31**), (**31A**) or (**31B**). The obtained spectral data, however, compatible only with the structures of (**31a**–**d**) and formulated as: *N*-(3-aryl-5-substituted-1,3,4-thiadiazol-2(3*H*)-ylidene)-4-(5-methyl-1-(*p*-tolyl)-1*H*-1,2,3-triazol-4-yl)thiazol-2-amine (**31a**–**d**) (Scheme 7).

**Scheme 8:**
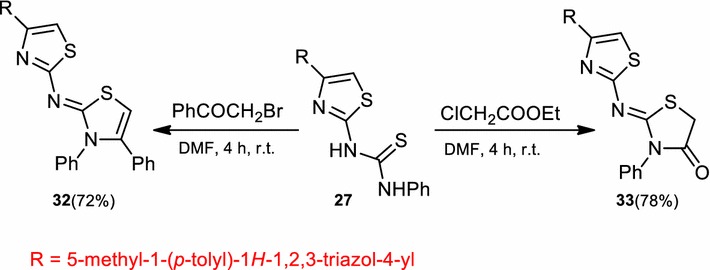
Synthesis of thiazole (**32**) and thiazolone (**33**)

Treatment of thiourea derivative (**27**) with ω-bromoacetophenone or ethyl chloroacetate in refluxing ethanol in the presence of triethylamine gave *N*-(3,4-diphenylthiazol-2(3*H*)-ylidene)-4-(5-methyl-1-(*p*-tolyl)-1*H*-1,2,3-triazol-4-yl)thiazol-2-amine (**32**) and 2-((4-(5-methyl-1-(*p*-tolyl)-1*H*-1,2,3-triazol-4-yl)-thiazol-2-yl)imino)-3-phenylthiazolidin-4-one (**33**), respectively (Scheme 8).

## Experimental section

### General methods

All melting points were determined on an electro thermal Gallen Kamp melting point apparatus (lain George, Calgary, Canda) and are uncorrected. IR (cm^−1^) spectra were recorded on KBr disk on a FTIR-8201 spectrophotometer (Shimadzu, Tokyo, Japan). ^1^H NMR and ^13^C NMR spectra were measured in deuterated dimethyl sulfoxide (DMSO-*d*6) using a Mercury VX-300 NMR spectrometer (Varian, Inc., Palo Alto, California 94304 USA). Mass spectra were recorded on a Shimadzu GCMS-QP1000 EX mass spectrometer (Tokyo, Japan) at 70 eV. Measurements of the elemental analysis were carried out at the Microanalytical Centre of Cairo University, Giza, Egypt. All reactions were followed by TLC (Silica gel, Merck, Kenilworth, NJ, USA). Hydrazonoyl halides were prepared as previously reported [32, 33].

#### Synthesis of sodium salt of 3-hydroxy-1-(5-methyl-1-(*p*-tolyl)-1*H*-1,2,3-triazol-yl)prop-2-en-1-one (**2**)

A solution of 1-(5-methyl-1-(*p*-tolyl)-1*H*-1,2,3-triazol-4-yl)ethan-1-one (**1**) [34], (5.4 g, 25 mmol) in ether (25 ml) was added to a mixture of sodium methoxide (1.4 g, 25 mmol) and ethyl formate (1.9 ml, 25 mmol) in dry ether (25 ml) while stirring in ice-bath at 0–5 °C for 2 h. The resulting solid was collected and washed with diethyl ether which afforded compound (**2**) that was used without crystallization, yield (76%).

#### Synthesis of 3-(dimethylamino)-1-(5-methyl-1-(*p*-tolyl)-1*H*-1,2,3-triazol-4-yl)prop-2-en-1-one (**6**)

A mixture of 1-(5-methyl-1-(*p*-tolyl)-1*H*-1,2,3-triazol-4-yl)ethane-1-one (**1**) (2.3 g, 0.1 mol) and *N,N*-dimethylformamide-dimethylacetal (11.9 g, 14 ml, 0.1 mol) in dry xylene (30 ml) was heated under reflux for 4 h. The hot solution evaporated to its half volume and then cooled. The resulting solid was collected and recrystallized from benzene to give the compound (**6**) as orange crystals. Yield: (83%); m.p. b135 °C. FT-IR (KBr, cm^−1^): 3041, 2965 (CH), 1688 (CO), 1645 (C=N), 1589 (C=C); ^1^H NMR (300 MHz, DMSO-d6): δ = 2.31 (s, 3H, CH_3_), 2.42 (s, 3H, CH_3_), 2.48 (s, 3H, CH_3_), 3.15 (s, 3H, CH_3_), 6.15 (d, 1H, *J* = 12 Hz, CH=), 7.76 (d, 1H, *J* = 12 Hz, CH=); 7.40–7.50 (m, 4H, ArH’s). Anal. Calcd. for C_15_H_18_N_4_O (270.34), C, 66.64; H, 6.71; N, 20.73. Found: C, 66.67; H, 6.69; N, 20.80.

#### Synthesis of pyrazolo[1,5-*a*]pyrimidines (**5a**–**c**), [1,2,4]triazolo[1,5-*a*]pyrimidine (**5d**), benzo [4,5]imidazo[1,2-*a*]pyrimidine (**5e**) and pyrido[2′,3′:3,4]pyrazolo[1,5-*a*]pyrimidine (**5f**)

##### Method A

A mixture of sodium salt (**2**) (1.32 g, 10 mmol) and the appropriate heterocyclic amines (**3a**–**f**) (10 mmol) in a solution of piperidinium acetate [piperidine (2.5 ml), water (5 ml) and acetic acid (2 ml)] was heated under reflux for 15 min, acetic acid (1.5 ml) was added to the reaction mixture while boiling, then the mixture was cooled and the resulting solid was collected and crystallized from the proper solvent gave (**5a**–**f**).

##### Method B

A mixture of compound (**6**) (1.35 g, 10 mmol), the appropriate heterocyclic amines (**3a**–**f**) (10 mmol) and ammonium acetate (0.77 g, 10 mmol) in acetic acid (20 ml) was heated under reflux for 4 h. The reaction mixture was cooled, after that, the resulting solid was collected and crystallized from the proper solvent and gave product identical in all aspects (m.p., mixed m.p., spectra) with the corresponding (**5a**–**f**), which was obtained in method A.

#### 7-(5-Methyl-1-(*p*-tolyl)-1*H*-1,2,3-triazol-4-yl)-2-phenylpyrazolo[1,5-*a*]pyrimidine (**5a**)

Yellow crystals from ethanol, yield (75%); m.p. 195–197 °C. FT-IR (KBr, cm^−1^): 2981 (CH); 1635 (C=N); 1566 (C=C). ^1^H NMR (300 MHz, CDCl_3_): δ = 2.50 (s, 3H, CH_3_) 2.65 (s, 3H, CH_3_), 6.82 (s, 1H, pyrazol H-4), 7.13 (d, 1H, *J* = 4 Hz, pyrimide H-5), 7.32–7.35 (m, 2H, ArH’s), 7.45–7.62 (m, 5H, ArH’s), 7.77–7.82 (m, 2H, ArH’s), 8.57 (d, 1H, *J* = 4 Hz, pyrimide H-6). ^13^C NMR (CHCl_3_) δ = 10.4, 20.6, 98.8, 111.2, 122.5, 127.4, 128.4, 128.8, 130.1, 131.8, 132.2, 133.4, 139.7, 141.2, 144.5, 146.4, 148.2, 152.3. Anal. Calcd. for C_22_H_18_N_6_ (366.43): C, 72.11; H, 4.95; N, 22.94. Found: C, 72.20; H, 4.80; N, 22.89.

#### 7-(5-Methyl-1-(*p*-tolyl)-1*H*-1,2,3-triazol-4-yl)-3-phenylpyrazolo[1,5-*a*]pyrimidine (**5b**)

Yellow crystals from ethanol, yield (75%); m.p. 230 °C. FT-IR (KBr, cm^−1^): 3028 (CH); 1635 (C=N); 1573(C=C). ^1^H NMR (300 MHz, CDCl_3_): 2.49 (s, 3H, CH_3_) 2.59 (s, 3H, CH_3_), 6.90–6.92 (d, 2H, *J* = *8* Hz, ArH’s), 7.10 (d, 1H, *J* = *8* Hz, pyrimidine H-5), 7.32–7.35 (m, 2H, ArH’s), 7.45–762 (m, 5H, ArH’s), 8.32 (s, 1H, pyrazole H-3), and 8.68 (d, 1H, *J* = 4 Hz, pyrimidine). Anal. Calcd. for C_22_H_18_N_6_ (366.43): C, 72.11; H, 4.95; N, 22.94. Found: C, 72.20; H, 4.80; N, 22.89.

#### 7-(5-Methyl-1-(p-tolyl)-1*H*-1,2,3-triazol-4-yl)-pyrazolo[1,5-a]pyrimidin-3-carbonitrile (**5c**)

Orange crystals from ethanol, yield (70%); m.p. 235–237 °C. FT-IR (KBr, cm^−1^): 3039, 2970 (CH); 2225 (CN); 1635 (C=N); 1573 (C=C). ^1^H NMR (300 MHz, CDCl_3_): δ = 2.49 (s, 3H, CH_3_) 2.54 (s, 3H, CH_3_), 7.26–7.59 (m, 5H, ArH^’^s), 8.95 (s, 1H, pyrazol H-3), and 8.84 (d, 1H, *J* = 4 Hz, pyrimidine H-6). ^13^C NMR in CHCl_3_ δ = 10.4, 20.6, 98.8, 52.4 (CN), 111.2, 11.3.1, 122.4, 128.4, 133.4, 135.1, 139.7, 141.2, 144.5, 146.4, 148.2, 155.3. Anal. Calcd. for C_17_H_13_N_7_ (315.39): C, 64.75; H, 4.16; N, 31.09. Found: C, 64.65; H, 4.26; N, 31.12.

#### 5-(5-Methyl-1-(*p*-tolyl)-1*H*-1,2,3-triazol-4-yl) [1,2,4]triazolo[1,5-*a*]pyrimidine (**5d**)

White crystals from acetic acid, yield (65%); m.p. 302 °C. FT-IR (KBr, cm^−1^): 3047, 2993 (CH); 1620 (C=N), 1577 (C=C). ^1^H NMR (300 MHz, DMSO-d6): δ = 2.07 (s, 3H, CH_3_) 2.49 (s, 3H, CH_3_), 6.62–6.63 (d, *J* = 4 Hz, 1H, pyrimidine H-5), 7.14–7.67(m, 4H, ArH,s), 8.27 (s, 1H, triazole), 9.27–9.28 (d, 1H, *J* = 4 Hz, pyrimidine H-6). Anal. Calcd. for C_15_H_13_N_7_ (291.32): C, 61.84; H, 4.50; N, 33.66. Found: C, 61.75; H, 4.40; N, 33.60.

#### 4-(5-Methyl-1-(*p*-tolyl)-1*H*-1,2,3-triazol-4-yl)benzo [4,5]imidazo[1,2-*a*]pyrimidine (5**e**)

Yellow crystals from ethanol, yield (65%); m.p. 200–202 °C. FT-IR (KBr, cm^−1^): 3047, 2981 (CH); 1635 (C=N); 1600 (C=C). ^1^H NMR (300 MHz, CDCl_3_): δ = 2.49 (s, 3H, CH_3_) 2.79 (s, 3H, CH_3_), 7.26–7.43 (m, 7H, ArH^’^s) 8.43–8.45(d, 1H, ArH), 8.80–8.82 (d 1H, *J* = 8 Hz, ArH), 9.65–9.66 (d, 1H, *J* = 8 Hz, pyrimidine H-6). MS (El), m/z (%): 338 (M-2,65), 323 (35), 304 (50), 275 (90), 262 (70), 249 (20), 221 (30), 132 (100), 91 (90), 77 (20), 65 (40). Anal. Calcd. for C_20_H_16_N_6_ (340.39), C, 70.57; H, 4.74; N, 24.69. Found: C, 70.64; H, 4.48; N, 24.58.

#### 8,10-Dimethyl-4-(5-methyl-1-(*p*-tolyl)-1*H*-1,2,3,-triazol-4-yl)pyrido[2′,3′:3,4]pyrazolo[1,5-*a*]pyrimidine (**5f**)

Yellow crystals from ethanol, yield (75%); m.p. 278–281 °C. FT-IR (KBr, cm^−1^): 3064, 2951, 2851 (CH); 1624 (C=N); 1597 (C=C). ^1^H NMR (300 MHz, DMSO-d6): δ = 2.44 (s, 3H, CH_3_), 2.51 (s, 3H, CH_3_), 2.60 (s, 3H, CH_3_), 2.88 (s, 3H, CH_3_), 6.98–7.00 (s, 1H, *J* = 8 Hz, pyridine H-3), 7.47–7.84 (m, 5H, ArH’s) and 8.89–8.87 (d, 1H, *J* = 8 Hz, pyrimidine H-6). ^13^C NMR (DMSO-d_6_ **)** δ = 10.4, 19.6, 20.6, 21.4, 101.2, 112.4, 114.8, 122.4, 125.7, 128.6, 130.4, 131.6, 139.4, 141.3, 145.5, 151.3, 153.2, 164.7. Anal. Calcd. for C_21_H_19_N_7_ (369.43), C, 68.28; H, 5.18; N, 26.54. Found: C, 68.20; H, 5.15; N, 26.45.

#### Synthesis of 5-methly-1-(*p*-tolyl)-1*H*-1,2,3-triazol-4-yl)(7-phenylpyrazolo[5,1-*c*]-[1,2,4]-triazin-3-yl)methanone (**10a**) and 5-methly-1-(*p*-tolyl)-1*H*-1,2,3-triazolo-4-yl)(8-phenyl pyrazolo[5.1-*c*][1,2,4]-triazin-3-yl)methanone (**10b**)

##### Method A

Dropwise addition of a solution of the appropriate diazonium salt of heterocyclic amines (**8a**) and (**8b**) (5 mmol) to a stirred mixture of sodium salt of (**2**) (1.25 g, 5 mmol), sodium acetate (0.65 g, 5 mmol) in ethanol (30 ml) at 0–5 °C. The solid so formed after 3 h and was collected, washed with water and recrystallized to give compound (**10a**) and, compound (**10b**), respectively.

##### Method B

A solution of the appropriate diazonium salt of heterocyclic amines (**8a**) or (**8b**) (5 mmol) were added dropwise while stirring a mixture of compound (**6**) (1.35 g, 5 mmol), sodium acetate (0.65 g, 5 mmol) in ethanol (30 ml) at 0–5 °C. The resulting solid so formed after 3 h and was collected, washed with water, and recrystallized to give product identical in all aspects (m.p., mixed m.p. and spectra) with the corresponding compound (**10a**) and compound (**10b**), which was obtained in method A.

#### 4-(5-Methyl-1-(*p*-tolyl)-1*H*-1,2,3-triazol-4-yl)-7-phenylpyrazolo[5,1-*c*][1,2,4]triazine (**10a**)

Brown crystals from ethanol, yield (75%); m.p. 215–217 °C. FT-IR (KBr, cm^−1^): 3058, 2969, 2922 (CH); 1681 (CO); 1639 (C=N); 1544 (C=C). ^1^H NMR (300 MHz, DMSO-d6): δ = 2.44 (s, 3H, CH_3_), 2.64 (s, 3H, CH_3_), 6.33 (s, 1H, pyrazole H-4), 7.32–7.34 (d, 2H, *J* = *8* Hz, ArH’s), 7.49–7.61 (m, 5H, ArH’s), 7.87–7.89 (d, 2H, *J* = *8* Hz, ArH’s) and 9.8 (s, 1H, triazine H-4). ^13^C NMR in DMSO-d6 δ = 10.4, 20.6, 101.1, 120.3, 121.4, 127.4, 128.5, 129.5, 130.2, 134.2, 134.6, 139.6, 142.4, 146.7, 153.1, 154.2. Anal. Calcd. for C_22_H_17_N_7_O (395.43): C, 66.82; H, 4.33; N, 24.80. Found: C, 66.89; H, 4.40; N, 24.75.

#### 4-(5-Methyl-1-(*p*-tolyl)-1*H*-1,2,3-triazol-4-yl)-8-phenylpyrazolo[5,1-*c*][1,2,4]triazine (**10b**)

Pale brown crystals from ethanol, yield (70%); m.p. 258–260 °C. FT-IR (KBr, cm^−1^): 3046,2919 (CH); 1675 (CO); 1646 (C=N); 1609 (C=C) ^1^H NMR (300 MHz, DMSO-d6): δ = 2.46 (s, 3H, CH_3_), 2.64 (s, 3H, CH_3_), 7.42–7.61 (m, 7H, ArH^’^s), 8.34–8.37 (d, 2H, *J* = *8* Hz, ArH,s), 9.24 (s, 1H, pyrazole H-3) and 10.19 (s, 1H, triazine H-4). ^13^C-NMR (DMSO-d_6_) δ = 10.4, 20.6, 102.3, 120.6, 121.3, 125.6, 126.8, 126.2,1 29.4, 130.2, 133.4, 134.8, 139.6, 142.5, 1146.7, 151.7, 154.8. Anal. Calcd. for C_22_H_17_N_7_O (395.43): C, 66.82; H, 4.33; N, 24.80. Found: C, 66.90; H, 4.37; N, 24.75.

#### Synthesis of 3-(5-methyl-1-(*p*-tolyl)-1*H*-1,2,3-triazol-4-y1)-3-oxo-2-(2-phenylhydrazono)propanal (**12a**) and 3-(5-methyl-1-(*p*-tolyl)-1*H*-1,2,3-triazol-4-y1)-3-oxo-2-(2-*p*-tolylhydrazono)propanal (**12b**)

##### Method A

Dropwise addition of a solution of the appropriate arenediazonium chloride (aniline and *p*-methylaniline) (5 mmol) to a stirred mixture of (**2**) (1.25 g, 5 mmol), sodium acetate (0.65 g, 5 mmol) in ethanol (30 ml) at 0–5 °C the solid so formed after 3 h and was collected and crystallized from ethanol to afford (**12a**) and (**12b**).

##### Method B

Dropwise addition of a solution of the appropriate arenediazonium chloride (aniline and *p*-methylaniline) (5 mmol) to a stirred mixture of (**6**) (1.35 g, 5 mmol), sodium acetate (0.65 g, 5 mmol) in ethanol (30 ml) at 0–5 °C. The solid so formed after 3 h then it was collected and crystallized from ethanol to give products identical in all aspects (m.p., mixed m.p., spectra) with corresponding compounds obtained from method A.

#### 3-(5-Methyl-1-(*p*-tolyl)-1*H*-1,2,3-triazol-4-y1)-3-oxo-2-(2-phenylhydrazono)propanal (**12a**)

Brown crystals from ethanol, yield (85%); m.p. 215–217 °C. FT-IR (KBr, cm^−1^): 3435 (NH); 2924 (CH); 1644 (C=N), ^1^H NMR (300 MHz, DMSO-d6): δ = 2.06 (s, 3H, CH_3_), 2.34 (s, 3H, CH_3_), 7.26–8.20 (m, 9H, ArH^’^s), 9.75 (s, 1H. CHO) and 14.39 (s, br.,1H, NH). Anal. Calcd. for C_19_H_17_N_5_O_2_ (347.38): C, 65.69; H, 4.93; N, 20.16. Found: C, 65.73; H, 4.84; N, 20.12.

#### 3-(5-Methyl-1-(*p*-tolyl)-1*H*-1,2,3-triazol-4-y1)-3-oxo-2-(2-*p*-tolyl)-hydrazono)propanal (**12b**)

Dark pink crystals from ethanol, yield (85%); m.p. 210–212 °C. FT-IR (KBr, cm^−1^): 3438 (NH); 2922 (CH), 1643 (C=C), ^1^H NMR (300 MHz, DMSO-d6): δ = 2.43 (s, 3H, CH_3_), 2.53 (s, 3H, CH_3_), 2.66 (s, 3H, CH_3_), 7.30–7.72 (m, 8H, ArH^’^s), 10.80 (s, 1H, CHO) and 13.9 (s, br., 1H, NH). Anal. Calcd. for C_20_H_19_N_5_O_2_ (361.41): C, 66.4; H, 5.30; N, 19.38. Found: C, 66.52; H, 5.38; N, 19.46.

#### Synthesis of 2-mercapto-6-(5-methyl-1-(*p*-tolyl)-1*H*-1,2,3-triazol-4-yl)nicotinonitrile (**14**)

##### Method A

A mixture of sodium salt (**2**) (1.25 g, 5 mmol) and 2-cyanothioacetamide (0.5 g, 5 mmol) in piperidine acetate [piperidine (2.5 ml), water (5 ml) and acetic acid (2 ml)] was heated under reflux for 15 min, acetic acid (1.5 ml) was added to the reaction mixture while boiling then the mixture was cooled and the resulting solid was collected and recrystallized from the proper solvent to give compound (**14**).

##### Method B

A mixture of (**6**) (1.35 g, 5 mmol) and cyanothioacetamide (0.5 g, 5 mmol) in ethanol (20 ml) and a catalytic amount of piperidine (10 ml) was heated under reflux for 4 h. After cooling, the resulting solid was collected and recrystallized from ethanol to afford compound **14** as brown crystals from ethanol, yield (65%); m.p. 262–265 °C. FT-IR (KBr, cm^−1^): 3074, 2962 (CH); 2218 (CN); 1573 (C=C). ^1^H NMR (300 MHz, DMSO-d6): δ = 2.43 (s, 3H, CH_3_), 2.61 (s, 3H, CH_3_), 5.87 (s, 1H, SH), 7.34–7.36 (d, 2H, *J* = 8 Hz, ArH’s), 7.52–7.54 (d, 2H, *J* = 8 Hz, ArH’s), 7.72–7.74 (d, 1H, *J* = 8 Hz, ArH’s), 8.39–8.41 (d, 1H, *J* = 8 Hz, ArH’s). ^13^C NMR (DMSO-d_6_) δ = 10.4, 20.6, 104.6, 116.5, 123.4, 125.8, 128.4, 139.7, 140.9, 143.8, 144.2, 147.2, 170.8, 173.8. MS (El, m/z (%): 308 (M + 1, 20), 294 (80), 278 (9), 264 (50), 237 (20), 219 (5), 177 (10), 144 (40), 132 (20), 91 (45), 80 (30), 64 (100). Anal. Calcd. for C_16_H_13_N_5_O (307.38), C, 62.52; H, 4.26; N, 22.78. Found: C, 62.57; H, 4.23; N, 22.85.

#### Synthesis of ethyl 3-amino-6-(5-methyl-1-(*p*-tolyl)-1*H*-1,2,3,-triazol-4-yl)thieno[2,3-*b*]pyridine-2-carboxylate (**15a**), 1-(3-amino-6-(5-methyl-1-(*p*-tolyl)-1*H*-1,2,3-triazol-4-y1)thino[2,3-*b*]pyridin-2-yl)-ethan-1-one (**15b**), 6-(3-amino–6-(5-methyl-1-(*p*-tolyl)-1*H*-1,2,3-triazal-4-yl)thieno[2,3-*b*]pyridin-2-yl)-(phenyl)methanone (**15c**), and 3-amino-6-(5-methyl-1-(*p*-tolyl)-1*H*-1,2,3-triazol-4-yl)thieno[2,3,-*b*]-pyridine-2-carbonitrile (**16**)

A mixture of compound (**14**) (2.1 g, 5 mmol), potassium hydroxide (0.28 g, 5 mmol) in *N,N*-dimethylformamide (10 ml) was stirred for 2 h then, the appropriate of ethyl chloroacetate, chloroacetone, ω-bromoacetophenone and chloroacetonitrile (5 mmol) was added while stirring. Stirring was continued for 2 h, the resulting solid was collected and crystallized from the proper solvent to afford compounds (**15a**–**c**), and (**16**) respectively.

#### Ethyl 3-amino-6-(5-methyl-1-(*p*-tolyl)-1*H*-1,2,3-triazol-4-yl)thieno[2,3-*b*]pyridine-2-carboxylate (**15a**)

Gray crystals from acetic acid, yield (65%); m.p. >300 °C. FT-IR (KBr, cm^−1^): 3460, 3355 (NH_2_); 3062, 2970 (CH), 1666 (CO); 1604 (C=C). ^1^H NMR (300 MHz, DMSO-d6): δ = 1.26 (t, 3H, *J* = *7* Hz, CH_2_CH_3_), 2.34 (s, 3H, CH_3_), 2.64 (s, 3H, CH_3_), 4.23 (q, 2H, *J* = *7* Hz, CH_2_CH_3_), 6.80 (s, br., 2H, NH_2_), 7.32–7.34 (d, 2H, *J* = *8* Hz, ArH’s), 7.52–7.54 (d, 2H, *J* = *8* Hz, ArH’s), 7.61–7.62 (d, 1H, *J* = *8* Hz, ArH’s),and 8.81–8.83 (d, 1H, *J* = *8* Hz, ArH); ^13^C NMR (DMSO-d_6_) δ = 10.4, 14.7, 20.6, 59.5, 105.7, 121.2, 123.2, 128.6, 133.8, 139.8, 140.7, 143.8, 44.2, 144.3, 149.7, 155.4, 166.1 Anal. Calcd. for C_20_H_19_N_5_O_2_S (393.47): C, 61.05; H, 4.87; N, 17.80 S, 8.1. Found: C, 61.15; H, 4.81; N, 17.76; S, 8.09.

#### 1-(3-Amino-6-(5-methyl-1-(*p*-tolyl)-1*H*-1,2,3-triazol-4-yl)thieno[2,3-*b*]pyridin-2-yl)ethanone (**15b**)

Brown crystals from acetic acid, yield (65%); m.p. 278–280 °C. FT-IR (KBr, cm^−1^): 3419, 3321 (NH_2_); 3092, 2920 (CH); 1675 (CO); 1593 (C=C). ^1^H NMR (300 MHz, DMSO-d6), δ = 2.35 (s, 3H, CH_3_), 2.49 (s, 3H, CH_3_), 2.62 (s, 3H, CH_3_), 5.79 (s, br., 2H, NH_2_), 7.32–7.34 (d, 2H, *J* = *8* Hz, ArH’s), 7.52–7.54 (d, 2H, *J* = *8* Hz, ArH’s), 7.70–7.72 (d, 1H, *J* = *8* Hz, ArH’s) and 8.71–8.73 (d, 1H, *J* = *8* Hz, ArH); ^13^C NMR (DMSO-d_6_) δ = 10.4, 20.6, 128.8, 120.4, 122.7, 123.6, 134.0, 139.8, 140.7, 143.5, 144.2, 149.4, 156.1, 190.9. Anal. Calcd. for C_19_H_17_N_5_OS (363.45): C, 62.79; H, 4.71; N, 19.27 S, 8.83. Found: C, 62.81; H, 4.71; N, 19.17; S, 8.75.

#### (3-Amino-6-(5-methyl-1-(*p*-tolyl)-1*H*-1,2,3-triazol-4-yl)thieno[2,3-*b*]pyridin-2-yl)(phenyl)methanone (**15c**)

Brown crystals from acetic acid, yield (65%); m.p. 220 °C. FT-IR (KBr, cm^−1^): 3402, 3286 (NH_2_); 3066, 2920 (CH); 1665 (CO); 1608 (C=C). ^1^H NMR (300 MHz, DMSO-d6): δ = 2.43 (s, 3H, CH_3_), 2.57 (s, 3H, CH_3_), 5.82 (s, br., 2H, NH_2_), 7.10–7.87 (m, 11H, ArH’s). Anal. Calcd. for C_24_H_19_N_5_OS (425.52), C, 67.74; H, 4.56; N, 16.46; S, 7.54. Found: C, 67.81; H, 4.60; N, 16.53; S, 7.62.

#### 3-Amino-6-(5-methyl-1-(*p*-tolyl)-1*H*-1,2,3,-triazol-4-yl)thieno[2,3,-*b*]pyridine-2-carbonitrile (**16**)

Brown crystals from acetic acid, yield (60%); m.p. 245 °C. FT-IR (KBr, cm^−1^): 3344, 3236 (NH_2_); 3058, 2923 (CH); 2194 (CN); 1639 (C=N); 1581 (C=C). ^1^H NMR (300 MHz, DMSO-d6): δ = 2.43 (s, 3H, CH_3_), 2.57 (s, 3H, CH_3_), 7.10–7.87 (m, 7H, ArH^’^s and NH_2_), 9.21–9.23 (d, 1H, *J* = 8 Hz, ArH). ^13^C NMR (DMSO-d_6_) δ = 10.4, 20.6, 93.8, 115.9, 118.6, 121.7, 125.1, 126.3, 126.7, 130.2, 133.2, 133.9, 138.7, 142.9, 147.9, 156.6. Anal. Calcd. for C_18_H_14_N_6_S (346.42), C, 62.41; H, 4.07; N, 24.26 S, 9.26. Found: C, 62.50; H, 4.17; N, 24.30; S, 9.36.

#### Synthesis of pyridine derivatives (**17**), (**18**) and (**20**–**22**)

A mixture of the appropriate ethyl acetoacetate, acetylacetone, ethyl cyanoacetate, benzoylacetonitrile, malononitrile (5 mmol), (**6**) (1.35 g, 5 mmol) and ammonium acetate (0.37 g, 5 mmol) in acetic acid (30 ml) was refluxed for 4 h, the resulting solid was collected and recrystallized from the proper solvent to give (**17**), (**18**), and (**20**–**22**), respectively.

#### Ethyl 2-methyl-6-(5-methyl-1-(*p*-tolyl)-1*H*-1,2,3,-triazol-4-yl)pyridine-3-carboxylate (**17**)

White crystals from ethanol, yield (75%); m.p. 190-192 °C. FT-IR (KBr, cm^−1^): 3039, 2920, 2800 (CH); 1774 (CO); 1647 (C=N); 1595 (C=C). ^1^H NMR (300 MHz, CDCl_3_): δ = 1.35 (t, 3H, *J* = 7 Hz, CH_2_CH_3_) 2.48 (s, 3H, CH_3_), 2.57 (s, 3H, CH_3_), 2.79 (s, 3H, CH_3_), 4.22 (q, 2H, *J* = 7 Hz, CH_2_CH_3_), 7.31–7.33 (d, 2H, *J* = *8* Hz, ArH’s), 7.52–7.54 (d, 2H, *J* = *8* Hz, ArH’s), 8.01–8.03 (d, 1H, *J* = *8* Hz, ArH’s), 8.45-8.47 (d, 1H, *J* = *8* Hz, ArH’s). ^13^C NMR (DMSO-d_6_) δ = 10.4, 14.4, 20.6, 25.5, 61.8, 121.5, 124.9, 126.4, 130.1, 133.2, 133.6, 134.7, 138.6, 143.1, 149.6, 158.7, 166.5. Anal. Calcd. for C_19_H_20_N_4_O_2_ (336.40): C, 67.84; H, 5.99; N, 16.66. Found: C, 67.90; H, 5.85; N, 16.56.

#### 1-(2-Methyl-6-(5-methyl-1-(*p*-tolyl)-1*H*-1,2,3-triazol-4-yl)pyridin-3yl)ethanone (**18**)

White crystals from benzene, yield (70%); m.p. 182-184 °C. FT-IR (KBr, cm^−1^): 2947, 2924 (CH); 1680 (CO); 1543 (CH). ^1^H NMR (300 MHz, CDCl_3_): δ = 2.48 (s, 3H, CH_3_), 2.63 (s, 3H, CH_3_), 2.79 (s, 3H, CH_3_), 2.82 (s, 3H, CH_3_). 7.32–7.34 (d, 2H, *J* = *8* Hz, ArH’s), 7.52–7.54 (d, 2H, *J* = *8* Hz, ArH’s), 7.86–7.88 (d, 1H, *J* = *8* Hz, ArH’s), 8.33–8.35 (d, 1H, *J* = *8* Hz, ArH’s). ^13^C-NMR (DMSO-d_6_) δ = 10.4, 20.6, 25.5, 27.6, 122.4, 125.1, 130.0, 130.5, 133.2, 133.7, 133.8, 138.7, 139.9, 151.2, 157.9, 200.1. MS [El, m/z (%)]: 306 (M^+^, 30), 289 (20), 278 (100), 263 (40), 220 (30), 205 (5) 160 (50), 144 (60), 117 (30), 91 (60), 77 (20), 65 (55). Anal. Calcd. for C_18_H_18_N_4_O (306.37): C, 70.57; H, 5.92; N, 18.29. Found: C, 70.43; H, 5.85; N, 18.35.

#### 6-(5-Methyl-1-(*p*-tolyl)-1*H*-1,2,3-triazol-4-yl)-2-oxo-1,2-dihydropyridine-3-carbonitrile (**20**)

Buff crystals from ethanol, yield (65%); m.p. 195 °C. FT-IR (KBr, cm^−1^): 3444 (NH); 3074, 2920, 2858 (CH); 2225 (CN); 1674 (CO); 1608 (C=N); 1585 (C=C). ^1^H NMR (300 MHz, CDCl_3_): δ = 2.48 (s, 3H, CH_3_) 2.70 (s, 3H, CH_3_), 7.09–7.11 (d, 1H, *J* = *8* Hz, ArH’s), 7.19–7.21 (d, 2H, *J* = *8* Hz, ArH’s), 7.44–7.16 (d, 2H, *J* = *8* Hz, ArH’s), 8.14–8.16 (d, 1H, *J* = *8* Hz, ArH’s), 11.65 (s, br., 1H, NH). Anal. Calcd. for C_16_H_13_N_5_O (291.31): C, 65.97; H, 4.50; N, 24.04. Found: C, 65.89; H, 4.59; N, 24.14.

#### 2-Amino-6-(5-methyl-1-(*p*-tolyl)-1*H*-1,2,3-triazol-4-yl)pyridine-3-carbonitrile (**21**)

White crystals from ethanol, yield (65%); m.p. >300 °C. FT-IR (KBr, cm^−1^): 3421, 3236 (NH_2_); 2924, 2854 (CH); 2220 (CN), 1643 (C=O); 1573 (C=C). ^1^H NMR (300 MHz, CDCl_3_): δ = 2.43 (s, 3H, CH_3_), 2.57 (s, 3H, CH_3_), 6.22 (s, 2H, NH_2_), 7.32–7.34 (d, 2H, *J* = *8* Hz, ArH’s), 7.52–7.54 (d, 2H, *J* = *8* Hz, ArH’s), 8.10–8.12 (d, 1H, *J* = *8* Hz, ArH’s), 8.56–8.58 (d, 1H, *J* = *8* Hz, ArH’s). Anal. Calcd. for C_16_H_14_N_6_ (290.33): C, 66.19; H, 4.86; N, 28.95. Found: C, 66.25; H, 4.75; N, 28.89.

#### 6-(5-Methyl-1-(*p*-tolyl)-1*H*-1,2,3-triazol-4-yl)-2-phenylnicotinonitrile (**22**)

Pale yellow crystals from ethanol, yield (65%); m.p. 270–273 °C. FT-IR (KBr, cm^−1^): 3059, 2918 (CH); 2200 (CN); 1608 (C=C). ^1^H NMR (300 MHz, DMSO-d6): δ = 2.42 (s, 3H, CH_3_), 2.62 (s, 3H, CH_3_), 7.32–7.54 (m, 9H, Ar’s), 7.68–7.88 (d, 1H, *J* = *8* Hz, ArH), 8.29–8.31 (d, 1H, *J* = *8* Hz, ArH). Anal. Calcd. for C_22_H_17_N_5_ (351.41): C, 75.19; H, 4.88; N, 19.93. Found: C, 75.16; H, 4.76; N, 19.82.

#### Synthesis of 4-(5-methyl-1-(*p*-tolyl)-1*H*-1,2,3-triazol-4-yl)thiazol-2-amine (**25**)

A mixture of 2-bromo-1-(5-methyl-1-(*p*-tolyl)-1*H*-1,2,3-triazol-4-yl)ethanone (**23**) (2.71 g, 0.01 mol) and thiourea (**24**) (0.76 g, 0.01 mol) in ethanol (50 ml) was heated under reflux for 30 min. The reaction mixture was poured on ice-cold water and drops of ammonia solution were added. The resulting solid so formed was collected and recrystallized from ethanol gave compound (**25**) as a white crystal, yield (93%); m.p. 192–194 °C. IR (KBr, cm^−1^): 3451, 3231 (NH_2_); ^1^H NMR (CDCl_3_): *δ* = 2.41 (s, 3H, CH_3_), 2.51 (s, 3H, CH_3_), 6.92–7.50 (m, 7H, ArH’s, NH_2_). ^13^C-NMR (DMSO-d_6_) δ = 10.4, 20.6, 119.7, 125.5, 128.9, 135.4, 139.6, 140.0, 140.7, 142.8, 173.8. MS: m/z = 271 (0.33), 248 (11), 223 (43), 213 (12), 212 (19), 169 (34), 141 (35), 108 (28), 79 (31), 77 (16), 70 (11). Anal. Calcd. For C_13_H_13_N_5_S (271.34): C, 57.54; H, 4.83; N, 25.81; S, 11.82. Found: C, 57.52; H, 4.86; N, 25.79; S, 11.84.

#### Synthesis of 4-(5-methyl-1-(*p*-tolyl)-1*H*-1,2,3-triazol-4-yl)-5-(aryldiazenyl)thiazol-2-amine (**26a**,**b**)

##### Method A

Arenediazonium chloride (5 mmol), which was prepared from aromatic amines (5 mmol), hydrochloric acid (6 N, 6 ml), and sodium nitrite (0.35 g, 5 mmol), then it was added dropwise with stirring to a cold solution of a mixture of (**25**) (1.35 g, 5 mmol) and sodium acetate trihydrate (1.3 g 10 mmol) in ethanol (50 ml). The resulting solid was collected and recrystallized from the proper solvent gave (**26a**,**b**).

##### Method B

A mixture of (**28**) (2 g, 5 mmol), thiourea (0.46 g, 6 mmol) and triethylamine (0.5 g, 0.72 ml, 5 mmol) in ethanol (25 ml) was heated under reflux for 2 h. The resulting solid was collected, washed with water, and crystallized from ethanol to give (**26a**).

#### 4-(5-Methyl-1-(*p*-tolyl)-1*H*-1,2,3-triazol-4-yl)-5-(phenyldiazenyl)thiazol-2-amine (**26a**)

A yellow crystals from ethanol, yield (65%); m.p. 218–220 °C. IR (KBr, cm^−1^): 3444, 3275 (NH_2_); ^1^H NMR (CDCl_3_): *δ* = 2.48 (s, 3H, CH_3_), 2.63 (s, 3H, CH_3_), 7.27–7.92 (m, 11H, ArH’s, NH_2_). ^13^C NMR (DMSO-d_6_) δ = 10.4, 20.6, 103.2, 118.1, 121.6, 127.4, 129.0, 130.2, 134.3, 139.8, 141.6, 141.9, 143.8, 155.0, 176.2. MS: m\z = 335 (15), 334 (21), 305 (10), 200 (61), 198 (35), 185 (13), 183 (15), 157 (14), 128 (14), 115 (16), 105 (25), 103 (45), 91 (21), 43 (99). Anal. Calcd. for C_19_H_17_N_7_S (375.45): C, 60.78; H, 4.56; N, 26.11; S, 8.54. Found: C, 60.85; H, 4.64; N, 26.21; S, 8.35.

#### Synthesis of 5-((4-chlorophenyl)diazenyl)-4-(5-methyl-1-(*p*-tolyl)-1*H*-1,2,3-triazol-4-yl)thiazol-2-amine (**26b**)

Yellow crystals from acetic acid gave, yield (65%); m.p. 168–170 °C. ^1^H NMR ((CD_3_)_2_SO): *δ* = 2.43 (s, 3H, CH_3_), 2.52 (s, 3H, CH_3_), 7.44–7.68 (m, 8H, ArH’s), 8.48 (s, 2H, NH_2_). Anal. Calcd. for C_19_H_16_ClN_7_S (409.90): C, 55.67; H, 3.93; N, 23.92; S, 7.82. Found: C, 55.52; H, 3.81; N, 24.10; S, 7.70.

#### Synthesis of 1-(4-(5-methyl-1-(*p*-tolyl)-1*H*-1,2,3-triazol-4-yl)thiazol-2-yl)-3-phenylthiourea (**27**)

A mixture of 4-(5-methyl-1-(*p*-tolyl)-1*H*-1,2,3-triazol-4-yl)thiazol-2-amine (**25**) (1.35 g, 5 mmol), phenyl isothiocyanate (0.6 ml, 5 mmol) and potassium hydroxide (0.28 g, 5 mmol) in DMF (10 ml) was stirred for 3 h. Then the mixture was poured on ice water containing HCl, the resulting solid was collected and crystallized from ethanol and gave white crystals, yield (75%); m.p. 200–202 °C. IR (KBr, cm^−1^): 3264 (NH), 3220 (NH), 1240 (C=S); ^1^H NMR ((CD_3_)_2_SO): *δ* = 2.42 (s, 3H, CH_3_), 2.58 (s, 3H, CH_3_), 7.20–7.65 (m, 10H, ArH’s), 10.95 (s, 1H, NH), 11.92 (s, 1H, NH); Ms: m/z = 406 (4), 390 (13), 370 (14), 297 (10), 284 (51), 271 (42), 252 (11), 242 (49), 210 (11), 200 (52), 183 (23), 168 (28), 156 (15), 144 (36), 125 (15), 115 (51), 105 (19), 102 (15), 91 (99), 85 (27), 77 (52), 69 (78), 65 (100), 52 (23), 45 (52). Anal. Calcd. for C_20_H_18_N_6_S_2_ (406.53): C, 59.09; H, 4.46; N, 20.67; S, 15.78. Found: C, 58.89; H, 4.64; N, 20.75; S, 15.84.

#### Synthesis of 2-[5-methyl-(*p*-tolyl)-1-*H*-1, 2, 3-trizol-4-yl]-2-oxo-*N*-phenylacetohydrazonoyl bromide (**28**)

A mixture of **(29**) (35.6 g, 0.1 mol) and *N*-nitrosoacetanilide [35] (10.4 g, 0.1 mol) in ethanol (100 ml) was stirred for 2 h at room temperature. The resulting solid was collected, washed with water and recrystallized from ethanol gave yellow crystals, yield (60%); m.p. 174–176 °C. IR (KBr, cm^−1^): 3441 (NH), 1651 (C=O), 1597 (C=N); ^1^H NMR (CDCl_3_): *δ* = 2.48 (s, 3H, CH_3_), 2.59 (s, 3H, CH_3_), 7.10–7.41 (m, 9H, ArH’s), 8.76 (s, 1H, NH); MS: m\z = 399 (22), 397 (22), 362 (18), 360 (55), 358 (56), 281 (25), 279 (50), 90 (18), 62 (15), 43 (99). Anal. Calcd. for C_18_H_16_BrN_5_O (398.26): C, 54.28; H, 4.05; N, 17.59. Found: C, 54.15; H, 4.14; N, 17.66.

#### Synthesis of dimethyl(2-(5-methyl-1-(*p*-tolyl)-1*H*-1,2,3-triazol-4-yl)-2-oxoethyl)sulfonium bromide (**29**)

A mixture of (**23**) (29.4 g, 0.1 mol) with dimethylsulfide (6.2 g, 0.1 mol) in ethanol (50 ml) was refluxed for 30 min. The reaction mixture was cooled to room temperature and then diluted with diethyl ether to complete precipitation. The resulting solid was collected and crystallized from ethanol to give white crystals, yield (78%); m.p. 134–135 °C.

#### Synthesis of 1,3,4-thiadiazole (**31a**–**d**), 2-((4-(5-methyl-1-(*p*-tolyl)-1*H*-1,2,3-triazol-4-yl)thiazol-2-yl)imino)-3-phenylthiazolidin-4-one (33) and N-(3,4-diphenylthiazol-2(3*H*)-ylidene)-4-(5-methyl-1-(*p*-tolyl)-1*H*-1,2,3-triazol-4-yl)thiazol-2-amine (**32**)

A mixture of 4-(5-methyl-1-(*p*-tolyl)-1*H*-1,2,3-triazol-4-yl)thiazol-2-amine (**25**) (1.35 g, 5 mmol), phenyl isothiocyanate (0.6 ml, 5 mmol) and potassium hydroxide (0.28 g, 5 mmol) in DMF (10 ml) was stirred for 3 h. then added appropriate hydrazonoyl chlorides (**30a**–**d**), or ethyl 2-chloroacetate (0.61 g, 5 mmol) or 2-bromo-1-phenylethanone (0.99 g, 5 mmol) and complete stirring 2 h, the resulting solid collected and recrystallized to give (**31a**–**d**), (**32**) and (**33**), respectively.

#### Ethyl 5-((4-(5-methyl-1-(*p*-tolyl)-1*H*-1,2,3-triazol-4-yl)thiazol-2-yl)imino)-4-phenyl-4,5-dihydro-1,3,4-thiadiazole-2-carboxylate (**31a**)

Yellow crystals from acetic acid, yield (74%); m.p. 253–254 °C. IR (KBr, cm^−1^): 1725 (C=O), 1597 (C=N), 1248, 1059 (CO); ^1^H NMR (CDCl_3_): *δ* = 1.42 (t, 3H, *J* = 7 Hz, CH_2_CH_3_), 2.48 (s, 3H, CH_3_), 2.80 (s, 3H, CH_3_), 4.49 (q, 2H, *J* = 7 Hz, CH_2_CH_3_), 7.27–7.59 (m, 9H, ArH’s), 8.55 (s, 1H, thiazole H-5). ^13^C-NMR (DMSO-d_6_) δ = 10.4, 14.5, 20.6, 62.9, 122.8, 123.7, 125.6, 127.9, 129.0, 130.1, 136.1, 139.6, 141.4, 143.8, 143.9, 148.2, 159.4, 161.1, 171.0. MS: m/z = 504 (10), 503 (37), 475 (58), 344 (32), 343 (15), 292 (16), 200 (100), 186 (24), 168 (33), 161 (23), 157 (13), 144 (22), 135 (11), 115 (20), 91 (72), 77 (47), 65 (23). Anal. Calcd. for C_24_H_21_N_7_O_2_S_2_ (503.60) C, 57.24; H, 4.20; N, 19.47; S, 12.73. Found: C, 57.31; H, 4.15; N, 19.57; S, 12.82.

#### Ethyl 5-((4-(5-methyl-1-(*p*-tolyl)-1*H*-1,2,3-triazol-4-yl)thiazol-2-yl)imino)-4-(p-tolyl)-4,5-dihydro-1,3,4-thiadiazole-2-carboxylate (**31b**)

Yellow crystals from acetic acid, yield (74%); m.p. 174–175 °C. IR (KBr, cm^−1^): 1677 (C=O), 1605 (C=N), 1244,1061 (CO); ^1^H NMR (CDCl_3_): *δ* = 1.36–1.46 (t, 3H, *J* = 7 Hz, CH_2_CH_3_), 2.33 (s, 3H, CH_3_), 2.45 (s, 3H, CH_3_), 2.80 (s, 3H, CH_3_), 4.45–4.52 (q, 2H, *J* = 7 Hz, CH_2_CH_3_), 7.13–7.57 (m, 8H, ArH’s), 8.55 (s, 1H, thiazole H-5); MS: m/z = 517 (5), 406 (11), 397 (15), 394 (10), 322 (44), 293 (26), 275 (14), 222 (13), 181 (12), 157 (14), 154 (14), 145 (16), 134 (15), 106 (100), 83 (50), 79 (56), 77 (46), 65 (54), 51 (35). Anal. Calcd. for C_25_H_23_N_7_O_2_S_2_ (517.63) C, 58.01; H, 4.48; N, 18.94; S, 12.39. Found: C, 58.12; H, 4.58; N, 19.10; S, 12.47.

#### 1-(5-((4-(5-methyl-1-(*p*-tolyl)-1*H*-1,2,3-triazol-4-yl)thiazol-2-yl)imino)-4-phenyl-4,5-dihydro-1,3,4-thiadiazol-2-yl)ethanone (**31c**)

Yellow crystals from acetic acid, yield (74%); m.p. 215–217 °C. IR (KBr, cm^−1^): 1649 (C=O), 1549 (C=N); ^1^H NMR (CDCl_3_): *δ* = 2.55 (s, 9H, CH_3_), 7.12–7.47 (m, 9H, ArH’s), 8.55 (s, 1H, thiazole H-5). ^13^C NMR (DMSO-d_6_) δ = 10.4, 20.6, 24.6, 122.8, 123.6, 125.1, 127.7, 127.9, 130.6, 136.1, 140.2, 142.1, 143.7, 147.1, 148.2, 171.1, 189.2. MS: m/z = 473 (7), 435 (15), 429 (12), 423 (12), 418 (14), 409 (11), 370 (94), 342 (46), 314 (25), 299 (12), 295 (13), 286 (17), 279 (17), 272 (30), 239 (12), 205 (17), 180 (13), 171 (35), 149 (30), 144 (38), 142 (30), 134 (54), 132 (13), 116 (38), 106 (35), 98 (22), 91 (100), 83 (44), 69 (42), 67 (33), 57 (52), 55 (80), 51 (32), 43 (44). Anal. Calcd. for C_23_H_19_N_7_OS_2_ (473.57) C, 58.33; H, 4.04; N, 20.70; S, 13.54. Found: C, 58.25; H, 3.90; N, 20.56; S, 13.49.

#### 1-(5-((4-(5-methyl-1-(*p*-tolyl)-1*H*-1,2,3-triazol-4-yl)thiazol-2-yl)imino)-4-(*p*-tolyl)-4,5-dihydro-1,3,4-thiadiazol-2-yl)ethanone (**31d**)

Yellow crystals from acetic acid, yield (74%); m.p. 211–212 °C. IR (KBr, cm^−1^): 1688 (C=O), 1601 (C=N); ^1^H NMR (CDCl_3_): *δ* = 2.35 (s, 3H, CH_3_), 2.46 (s, 3H, CH_3_), 2.66 (s, 3H, CH_3_), 2.81 (s, 3H, CH_3_), 7.16–7.61 (m, 8H, ArH’s), 8.55 (s, 1H, thiazole H-5); MS: m/z = 491 (23), 488 (11), 487 (36), 459 (58), 357 (11), 285 (19), 276 (23), 201 (24), 200 (100), 186 (24), 175 (18), 168 (27), 157 (13), 142 (21), 132 (17), 115 (14), 105 (16), 91 (57), 65 (19). Anal. Calcd. for C_24_H_21_N_7_OS_2_ (487.60) C, 59.12; H, 4.34; N, 20.11; S, 13.15. Found: C, 59.21; H, 4.43; N, 20.25; S, 13.22.

#### *N*-(3,4-diphenylthiazol-2(3*H*)-ylidene)-4-(5-methyl-1-(*p*-tolyl)-1*H*-1,2,3-triazol-4-yl)thiazol-2-amine (**32**)

Yellow crystals from acetic acid, yield (72%); m.p. 270–272 °C. IR (KBr, cm^−1^): 3114 (=CH); ^1^H NMR (CDCl_3_): *δ* = 2.42 (s, 3H, CH_3_), 2.78 (s, 3H, CH_3_), 6.38 (s, 1H, CH), 7.15–7.52 (m, 14H, ArH’s), 8.67 (s, 1H, thiazole H-5); MS: m/z = 506 (8), 505 (23), 477 (25), 294 (44), 278 (21), 275 (23), 251 (11), 200 (32), 180 (12), 168 (13), 134 (21), 115 (15), 105 (38), 91 (69), 77 (100), 65 (47), 51 (28), 45 (15). Anal. Calcd. for C_28_H_22_N_6_S_2_ (506.64): C, 66.38; H, 4.38; N, 16.59; S, 12.66. Found: C, 66.27; H, 4.45; N, 16.67; S, 12.72.

#### 2-((4-(5-Methyl-1-(*p*-tolyl)-1*H*-1,2,3-triazol-4-yl)thiazol-2-yl)imino)-3-phenylthiazolidin-4-one (**33**)

Pink crystals from acetic acid, yield (78%); m.p. 285–287 °C. IR (KBr, cm^−1^): 1730 (C=O); ^1^H NMR ((CD_3_)_2_SO): *δ* = 2.43 (s, 3H, CH_3_), 2.67 (s, 3H, CH_3_), 3.96 (s, 2H, CH_2_), 7.37–7.77 (m, 9H, ArH’s), 8.67 (s, 1H, thiazole H-5); MS: m/z = 447 (7), 446 (26), 418 (100), 201 (18), 200 (91), 186 (22), 168 (30), 144 (19), 142 (24), 115 (20), 91 (48), 77 (42), 65 (21); Anal. Calcd. for C_22_H_18_N_6_OS_2_ (446.55) C, 59.17; H, 4.06; N, 18.82; S, 14.36. Found: C, 59.17; H, 4.06; N, 18.82; S, 14.36.

## Conclusions

New series of pyrazolo[1,5-*a*]pyrimidines, pyrazolo[5,1-*c*]triazines, thieno[2,3-b]pyridines and polysubstituted pyridines containing the 1,2,3,-triazole moiety were synthesized via reactions of sodium 3-(5-methyl-1-(*p*-tolyl)-1*H*-1,2,3-triazol-4-yl)-3-oxoprop-1-en-1-olate with the appropriate heterocyclic amines and its diazonium salt. In addition, 1,3,4-thiadiazoles and, 1,3-thiazoles were acquired in a decent yield via the reaction of substituted thiourea with the appropriate hydrazonoyl chlorides and halogenated ketenes.

## Abbreviations

COX-2: cyclooxygenase-2
CNS: the central nervous system
HMG-CoA: the enzyme 3-hydroxy-3-methyl-glutaryl-co-enzyme A
KDR: kinase insert domain receptor
PDE: a phosphodiesterase
MW: molecular weight
*TLC*: thin layer chromatography
